# Spatio-temporal modeling of human leptospirosis prevalence using the maximum entropy model

**DOI:** 10.1186/s12889-023-17391-z

**Published:** 2023-12-16

**Authors:** Reza Shirzad, Ali Asghar Alesheikh, Mojtaba Asgharzadeh, Benyamin Hoseini, Aynaz Lotfata

**Affiliations:** 1 Department of Geospatial Information System, Faculty of Geodesy and Geomatics Engineering, K. N. Toosi University of Technology, Tehran, Iran; 2 Pharmaceutical Research Center, Pharmaceutical Technology Institute, Mashhad University of Medical Sciences, Mashhad, Iran; 3 Department of Medical Informatics, Faculty of Medicine, Mashhad University of Medical Sciences, Mashhad, Iran; 4 School Of Veterinary Medicine, Department of Pathology, Microbiology, and Immunology, University of California, Davis, USA

**Keywords:** Leptospirosis incidence, Spatial-temporal modeling, SaTScan, MaxEnt

## Abstract

**Background:**

Leptospirosis, a zoonotic disease, stands as one of the prevailing health issues in some tropical areas of Iran. Over a decade, its incidence rate has been estimated at approximately 2.33 cases per 10,000 individuals. Our research focused on analyzing the spatiotemporal clustering of Leptospirosis and developing a disease prevalence model as an essential focal point for public health policymakers, urging targeted interventions and strategies.

**Methods:**

The SaTScan and Maximum Entropy (MaxEnt) modeling methods were used to find the spatiotemporal clusters of the Leptospirosis and model the disease prevalence in Iran. We incorporated nine environmental covariates by employing a spatial resolution of 1 km x 1 km, the finest resolution ever implemented for modeling Human Leptospirosis in Iran. These covariates encompassed the Digital Elevation Model (DEM), slope, displacement areas, water bodies, and land cover, monthly recorded Normalized Difference Vegetation Index (NDVI), monthly recorded precipitation, monthly recorded mean and maximum temperature, contributing significantly to our disease modeling approach. The analysis using MaxEnt yielded the Area Under the Receiver Operating Characteristic Curve (AUC) metrics for the training and test data, to evaluate the accuracy of the implemented model.

**Results:**

The findings reveal a highly significant primary cluster (*p*-value < 0.05) located in the western regions of the Gilan province, spanning from July 2013 to July 2015 (*p*-value < 0.05). Moreover, there were four more clusters (*p*-value < 0.05) identified near Someh Sara, Neka, Gorgan and Rudbar. Furthermore, the risk mapping effectively illustrates the potential expansion of the disease into the western and northwestern regions. The AUC metrics of 0.956 and 0.952 for the training and test data, respectively, underscoring the robust accuracy of the implemented model. Interestingly, among the variables considered, the influence of slope and distance from water bodies appears to be minimal. However, altitude and precipitation stand out as the primary determinants that significantly contribute to the prevalence of the disease.

**Conclusions:**

The risk map generated through this study carries significant potential to enhance public awareness and inform the formulation of impactful policies to combat Leptospirosis. These maps also play a crucial role in tracking disease incidents and strategically directing interventions toward the regions most susceptible.

**Supplementary Information:**

The online version contains supplementary material available at 10.1186/s12889-023-17391-z.

## Introduction

The worldwide impact of Leptospirosis on human health is substantial, with an estimated annual burden of 1.03 million cases and 58,900 fatalities [1, 2]. This disease is transmitted by the *spirochaete bacterium Leptospira* and stands as a prevalent zoonotic disease, significantly contributing to global public health concerns [3]. While human-to-human transmission of Leptospirosis is infrequent, the bacteria can be transmitted to humans through sources such as contaminated water, soil, or direct contact with infected animals [4]. The symptoms of Leptospirosis disease encompass fever, headache, jaundice, kidney failure, meningitis, and respiratory failure, potentially leading to fatal outcomes in some cases [1]. Given the likeness of its initial symptoms to the flu and common cold, Leptospirosis is commonly susceptible to misdiagnosis. Furthermore, the treatment of patients imposes substantial financial burdens on governments and healthcare systems within countries [1]. The World Health Organization (WHO) has characterized Leptospirosis as a “neglected disease of global significance,“ underscoring the need for increased focus and research on its worldwide prevalence [5].

Leptospirosis disease is endemic in various tropical, subtropical, and temperate regions. Its prevalence can surge considerably in vulnerable areas, especially in the aftermath of heavy rains and flooding [6]. The prevalence of the disease in a particular region is shaped by intricate interplays among environmental factors and socioeconomic conditions of the residents. These encompass climate, topography, land cover, surface water levels, occupation, as well as the presence of domestic and wild animal populations, notably rodents, which wield a significant influence [7].

While the use of spatial analysis techniques to identify infectious disease risk areas has increased exponentially over the last two decades [6, 8–11], there remains a lack of substantial research into the spatial distribution of Leptospirosis. Chadsuti et al. (2022) used spatial autocorrelation analysis in conjunction with local indicators of spatial association (LISA) to identify disease clusters in Thailand. They also used a generalized linear mixed model (GLMM) to determine the most important environmental factor for Leptospirosis disease. Their findings highlighted a strong correlation between occurrences of Leptospirosis disease and flooded areas [12]. Mayfield et al. (2018) employed geographically weighted logistic regression (GWL) to create a Leptospirosis risk map for Fiji in the Oceania region. Their model incorporated Leptospirosis cases within Fiji, considering variables such as cattle density, distance from the river, poverty rate, urban or rural living conditions, and maximum rainfall during the wet month. Notably, their findings indicated that the geographically weighted logistic regression method demonstrated greater efficacy compared to the standard logistic regression approach [13]. In Mexico, Sokani Sanchez-Montes et al. (2015) utilized the Genetic Algorithm for Rule-set Prediction (GARP) technique, which is an ecological niche modeling method, to predict the distribution of Leptospirosis. They used temperature and precipitation-derived data as inputs for their model [14]. White et al. (2017) investigated the correlation between environmental and socioeconomic factors and Leptospirosis cases in animals within the United States. They employed machine learning techniques to spatially forecast the occurrence of the disease [15]. Another study [16] predicted that biological control would have the greatest impact on lowering Leptospirosis morbidity. Zhao et al. (2016) established a statistical connection between the occurrence of Leptospirosis and nine environmental and socio-economic determinants in China, employing MaxEnt and Logistic regression models. Both models displayed robust predictive performance, achieving AUC values of 0.95 and 0.96, respectively. Notably, the geographic distribution of Leptospirosis in China is primarily influenced by annual mean temperature and annual total precipitation [3].

Unlike earlier research [17–21], which predominantly concentrated on Leptospirosis cases in northern regions owing to the disease’s endemic nature, this study broadens its scope to cover the entire country. It incorporates socio-environmental factors to cultivate a more comprehensive understanding of Leptospirosis distribution. Specifically, the integration of factors such as land cover and displacement areas are undertaken to enhance the accuracy of the geographical distribution likelihood map for Leptospirosis in Iran. These considerations, often overlooked in prior research [18, 19], are crucial for assisting public health professionals in identifying areas of heightened risk.

In terms of methodology, prior research [13, 17, 19] primarily employed Geographically Linear Weighted Regression. However, our study utilizes MaxEnt as a presence-only method, enabling the incorporation of numerous environmental variables. This approach accommodates both linear and nonlinear relationships, proving effective even in cases of limited sample sizes.

Over a decade-long timeframe, our primary aim is to conduct a robust statistical investigation into the patterns and trends of a disease within Iran. Using the MaxEnt modeling technique, this research seeks to explore the spatial and temporal dynamics of Leptospirosis across the nation. Beyond conventional observations, this study considers diverse demographic and occupational groups, along with environmental variables, with the goal of providing a comprehensive understanding of the disease’s change over time.

## Materials and methods

### Study area

Covering the entire expanse of Iran, the study area extends between latitudes 24° and 40° N, and longitudes 44° and 64° E. Iran’s geographic layout is demarcated into 31 provinces, accommodating a population exceeding 81 million people across its vast 1.65 million km^2^ territory. The nation showcases notable climatic and topographical diversity, spanning from areas situated a few tens of meters below sea level to altitudes surpassing 5,600 m. This diversity is further emphasized by annual precipitation variations ranging from less than 100 millimeters (mm) to around 2,000 mm, coupled with temperature fluctuations ranging from − 10 to + 50 degrees Celsius. Iran’s climate spectrum ranges from arid to subtropical zones [22].

### Leptospirosis cases as the outcome variable

During the period from 2009 to 2018, the Ministry of Health and Medical Education of Iran documented a cumulative count of 3,433 cases across 17 out of the 31 provinces as referral centers for Leptospirosis. The incidence rate is the ratio of newly infected people to the population at risk at the beginning of the observation period that for Leptospirosis is about 4.8 per 100,000 people in the study period in Iran. In an effort to eliminate redundancy, a comprehensive investigation was conducted for all Leptospirosis cases. For geocoding confirmed instances, the latitude and longitude of patients’ residential addresses were utilized, facilitated by Google Earth Pro 9.0 (Google Inc.). Cases lacking patients’ residential addresses were excluded from the analysis as potential errors.

### Covariates

The Digital Elevation Model (DEM), Normalized Difference Vegetation Index (NDVI), precipitation, mean temperature, maximum temperature, slope, displacement areas, water areas, and land cover are nine environmental covariates provided by various sources. The selection of these covariates is grounded in data availability and their established utility in disease spatial distribution modeling, as evidenced by their use in existing literature as influential factors. We used 1 km*1km resolution for environmental data as the best spatial resolution ever used in Iran for Leptospirosis modeling because locating the exact location of bacteria transmission to the patient is difficult.

To explore the impact of meteorological variables on Leptospirosis distribution, temperature and precipitation data spanning the years 2009 to 2018 were sourced from the Meteorological Organization’s website in Iran [23]. This dataset encompassed average, maximum, and minimum temperatures in degrees Celsius, along with total monthly rainfall in millimeters. The information was extracted from more than 350 synoptic stations dispersed across the country.

The mean NDVI is calculated for the years 2009 to 2018, and a 10-year mean NDVI map using the Moderate Resolution Imaging Spectroradiometer (MODIS) [24]. Monthly 1 km*1km images were employed to compute the average NDVI map. NDVI values, which span from − 1 to + 1, signify greater green vegetation density with higher values. The NDVI calculation involved merging Near-Infrared (NIR) and Red spectral bands from the MODIS product, conducted through the ENVI 5.3 software.

To explore the influence of land cover on disease occurrence, land cover maps from GlobeCover Land Cover version 2.3 were extracted. Utilizing the Medium Resolution Imaging Spectrometer (MERIS) with a resolution of 300 m, a comprehensive land cover map featuring 18 classes (see Additional file 1: Appendix Table A.1) was generated for all landmasses. These maps were sourced from the European Space Agency [25].

Water area maps were generated by identifying water bodies like rivers and lakes. Through a buffer analysis utilizing a 1 km radius, the shapefile underwent conversion to a raster format using the “polygon to raster” tool. The pixel values within the water areas map correspond to the Euclidean distance from water bodies within the study region, spanning from 0 to 190 km. Consequently, pixel values of “0” denote water bodies, while higher values indicate the distance in kilometers from the nearest water body within the study vicinity.

Additionally, the displacement areas variable has been developed to investigate whether bacteria transmission is associated with the movement of contaminants through vehicle droppings, or the transfer of soil and vegetation to different areas. The displacement map was created using data from roads, land and sea border crossings, and airports. Similar to the process for the water areas map, this map was generated. The pixel values within this map indicate the distance from roads, land and sea border crossings, and airports in kilometers, spanning from 0 to 116 km within our study area.

The DEM map of Iran is extracted from the Advanced Spaceborne Thermal Emission and Reflection (ASTER) satellite images [26]. The Ministry of Economy, Trade, and Industry of Japan (METI) and NASA released this global dataset in 2009, covering approximately 99% of the Earth’s land surface with a pixel size of 30 m and vertical accuracy of 20 m with 95% confidence. The slope map is then created from that.

### Methods

**Fig. 1:**
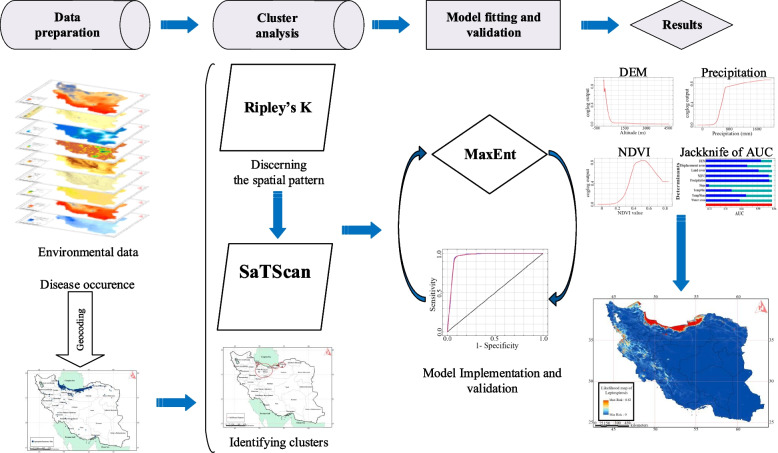
Study flow diagram for producing likelihood map of Leptospirosis in Iran

The overall framework of this study included data preparation, cluster analysis, the model implementation and validation, and producing likelihood map of Leptospirosis in Iran (Fig. 1). We introduce the details of each step in the following sections:

#### Data preparation

For the preparation of meteorological layers, a point pattern map was created, utilizing station coordinates and their corresponding temperature and rainfall values. Subsequently, the reverse distance interpolation (IDW) technique was applied, yielding a continuous map with a resolution of 1 km*1km. All maps were generated utilizing a primary raster mask map with a resolution of 1 km*1km as a template, serving as input maps for MaxEnt. The utilization of this mask map ensures uniformity in terms of pixel number, size, and location across all maps, ensuring complete overlap of identical pixels in different maps. Furthermore, to assess collinearity, a Pearson correlation analysis was employed. Variables exhibiting collinearity exceeding 0.7 were excluded from the analysis to prevent multicollinearity issues [3].

#### Cluster analysis

To analyze spatial pattern of disease distribution, the study utilized the Ripley’s K [27] method. This function can help researchers and analysts discern the underlying spatial processes and patterns that might be present in a dataset. Additionally, the Monte Carlo simulation was incorporated to address edge effects in Ripley’s K [27]. This technique involves simulating random sample point distributions and comparing them with the actual distribution. In this study, this process was iterated 999 times to achieve a robust 99% confidence level in the results. Ripley’s K function is often used in combination with its spatial counterpart, the L function (L(r)), and the pair correlation function (PCF), to gain a deeper understanding of point patterns in various fields like ecology, epidemiology, criminology, and more. Ripley’s K function and the L function are often used together to obtain a comprehensive understanding of the point pattern [27]. While the K function emphasizes interactions between points at various distances, the L function focuses on the distribution of nearest neighbor distances. Subsequently, the spatial scan statistics (SaTScan) method was applied to identify spatio-temporal clusters. In SaTScan, the space-time permutation model was utilized. This model was chosen due to its ability to identify disease clusters without the necessity of demographic or disease control station data [28]. Space-time scan statistics are a prevalent tool employed to detect geographical clusters in disease surveillance and the transmission of infectious agents [29]. This approach is particularly valuable for time-periodic prospective surveillance, involving repetitive analysis over specific time intervals, such as monthly or yearly assessments.

The spatial scan statistic employs a varying-shaped window, often resembling a circle, to traverse a geographic area as it identifies potential clusters. The likelihood for each candidate cluster is computed by considering observed and expected case numbers within and outside the defined region. Within SaTScan, there exist different models for cluster identification. In this study, the “space-time permutation” model was chosen due to its unique advantage in detecting spatiotemporal clusters solely using disease occurrence points and temporal data, unlike other SaTScan’s models that require data on disease control centers or precise demographic information about the population at risk [30], which is lacking in our study area, the selected model operates effectively without these prerequisites.

The alternative hypothesis for each window is that there is an elevated risk within the window as compared to the outside. The likelihood function for a scanning window with specific location and size is proportional to [31]:

1 $${\left(\frac{C}{E\left(c\right)}\right)}^{c}{\left(\frac{C-c}{C-E\left(c\right)}\right)}^{C-c}I\left(\right)$$

where C is the total number of cases, c is the observed number of cases within the window and E[c] is the covariate adjusted expected number of cases within the window under the null-hypothesis, C-E[c] is the expected number of cases outside the window and I() is an indicator function. I() will equal to 1 if SaTScan is set to scan only for clusters with high rates. The window with the maximum likelihood considered as the most likely cluster that is least likely to have occurred by chance [31]. The *p*-value is calculated through Monte Carlo hypothesis testing [32], by comparing the rank of the maximum likelihood from the real data set with the maximum likelihoods from the random data sets I. For a comprehensive understanding of the mathematical framework, refer to Kulldorff’s work [31].

#### Modeling

The Maximum Entropy algorithm (MaxEnt) stands as a prevalent machine learning modeling technique, rooted in the second law of thermodynamics [33]. It is designed to estimate the probability distribution of Leptospirosis disease by determining the probability distribution with the highest entropy [33]. Here, as usual, the entropy of a distribution p on X is defined to be [34]:

2 $$H\left(p\right)=-{\sum }_{x\in X}p\left(x\right)*\text{ln}\ p\left(x\right)$$

X is a set of discrete grid cells, each representing a locality where the species has been observed. Also, a set of environmental variables are defined on X. In the context of the Maximum Entropy Model, we want to find the distribution $p\left(x\right)$ that maximizes entropy while satisfying a set of constraints which formulated as an optimization problem. These constraints can be defined in terms of expected values of certain functions $f\left(x\right)$:

3 $$E\left[f\left(x\right)\right]=\sum p\left(x\right)*f\left(x\right)$$

Where $E\left[f\left(x\right)\right]$ is the expected value of the function $f\left(x\right)$ under the distribution $p\left(x\right)$. To solve this optimization problem, Lagrange multipliers λ for each constraint is introduced. Here is the Lagrangian function $L\left(p,\lambda \right)$:

4 $$L\left(p,\lambda \right)=-\sum p\left(x\right)*\text{ln}\ p\left(x\right)+\sum \lambda *(E\left[f\left(x\right)\right]-\sum p\left(x\right)*f\left(x\right))$$

The distribution $p\left(x\right)$ that maximizes the entropy under the given constraints is found by taking the derivative of the Lagrangian function $L\left(p,\lambda \right)$ with respect to $p\left(x\right)$, setting it to zero:

5 $$\partial L/\partial p\left(x\right)=0$$

This leads to:

6 $$p\left(x\right)=(1/\sum {e}^{(\sum \lambda *f\left(x)\right)})*{e}^{\left(\sum \lambda *f\left(x\right)\right)}$$

Finally, among all possible distributions consistent with the constraints, the MaxEnt distribution is the one that makes the fewest assumptions beyond the constraints [34]. This method finds widespread application in spatial distribution modeling, delivering not only high accuracy but also circumventing the need for absence data (in this instance, the lack of Leptospirosis locations in a region). In this method, instead of absence points, pseudo-absence points are randomly generated by the model. For modeling purposes, 60% of the data was randomly selected, while the remaining portion was reserved for model testing.

To assess the MaxEnt model’s performance, the Area Under the Receiver Operating Characteristic Curve (AUC) was utilized as a metric [35]. An ROC curve charts the true positive rate against the false positive rate across different classification thresholds in a classification problem. The AUC quantifies the area under this curve comparing disease occurrences to randomly generated background locations within the region [34]. In cases involving presence-only models, an AUC greater than 0.75 indicates the model’s predictions based on presence-only cases are acceptably accurate [36].

Finally, two additional methods, namely the Jack Knife’s test [34] and covariates’ percent contribution produced by MaxEnt, were employed to ascertain the significance of variables in the modeling procedure. The Jack Knife’s test assesses variables by executing the algorithm twice: once with the specific determinant and once with all determinants except the variable of interest. This process helps gauge the effectiveness of the variable in comparison to the final model. On the other hand, the contribution percentage method provides insight into the relative impact of variables on the model’s output [34].

## Results

### Leptospirosis cases summary

**Fig. 2:**
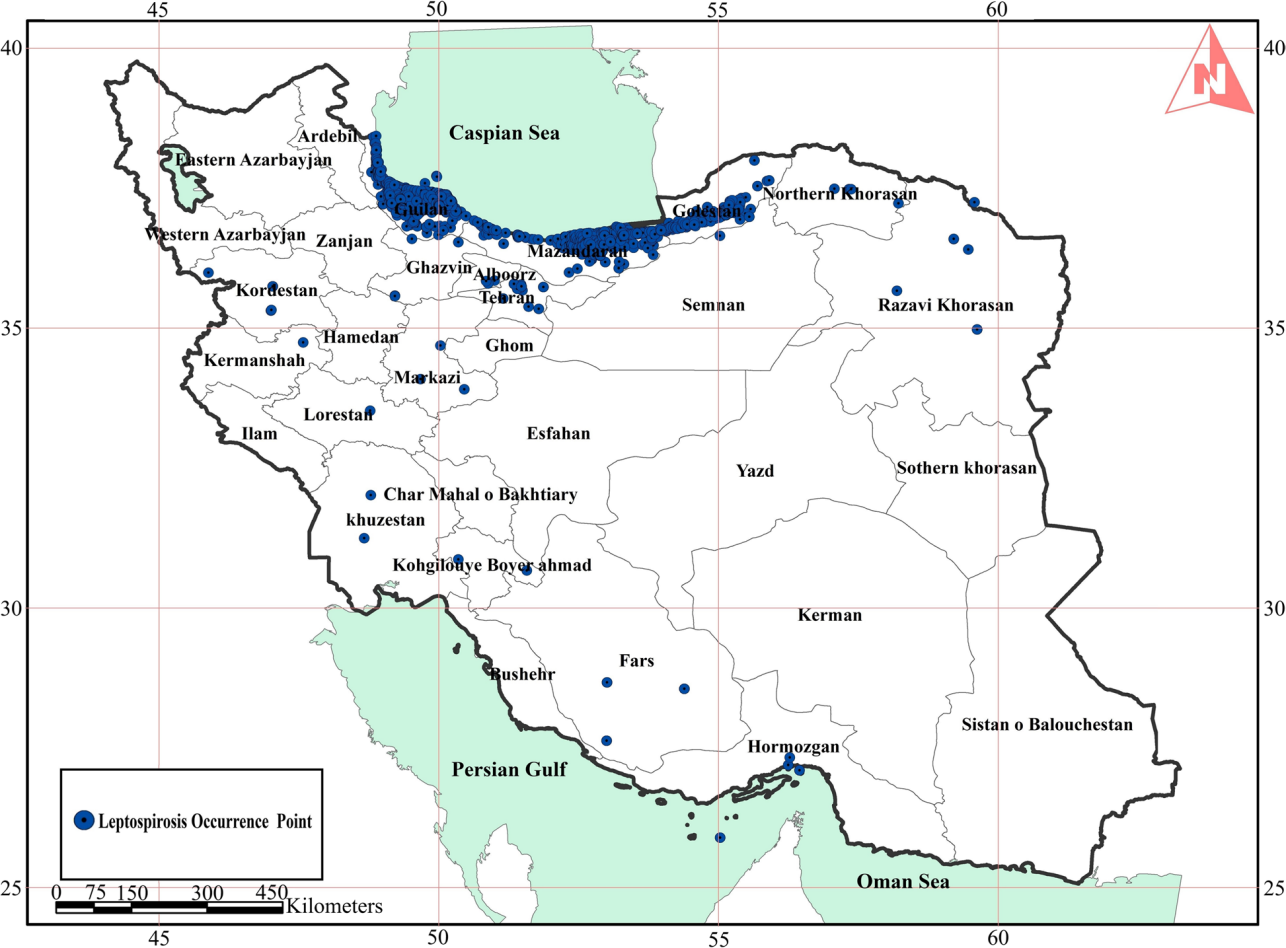
Leptospirosis occurrence cases in Iran from 2009–2018

Figure 2 shows the prevalence of leptospirosis in Iran from 2009 to 2018. As shown in this figure prevalence of leptospirosis is highest in northern region. Also, some cases are recorded near Tehran and adjacent cities.

Table 1 presents an overview of Leptospirosis cases in Iran spanning from 2009 to 2018, categorized by province, gender, age, and occupation. Notably, over 50% of individuals within the “other” subcategory of the occupational group reported exposure to contaminated water through activities like swimming in rivers or consuming non-tap water. In terms of gender, males face a higher risk, and individuals within the age range of 35–64 are particularly susceptible.

**Table 1:** Statistics of Leptospirosis in Iran from 2009–2018

**Province**	**Province**	**Incidence Rate (per 100,000 pop)**											
		**Total (2009-2018)**	**2009**	**2010**	**2011**	**2012**	**2013**	**2014**	**2015**	**2016**	**2017**	**2018**	***p*-value**
	Gilan	94.81	9.84	17.82	6.93	3.43	7.98	8.67	13.02	17.17	4.51	5.44	<0.0001
	Mazandaran	25.37	0.84	5.16	0.84	0.91	1.36	1.40	1.98	5.71	3.41	3.76	<0.0001
	Golestan	13.9	1.81	3.62	0.90	0.68	1.41	0.73	0.96	1.86	1.41	0.51	<0.0001
	Tehran	0.09	0.00	0.02	0.02	0.01	0.01	0.00	0.02	0.00	0.01	0.00	<0.0001
	Khorasan Shomali	0.69	0.00	0.00	0.00	0.00	0.00	0.00	0.20	0.00	0.39	0.10	<0.0001
	Fars	0.11	0.00	0.07	0.02	0.00	0.00	0.00	0.00	0.02	0.00	0.00	<0.0001
	Khorasan Razavi	0.07	0.00	0.00	0.00	0.00	0.00	0.00	0.04	0.04	0.00	0.00	<0.0001
	Qazvin	0.33	0.00	0.00	0.00	0.00	0.00	0.00	0.00	0.00	0.11	0.22	<0.0001
	Kordestan	0.27	0.00	0.00	0.00	0.00	0.00	0.07	0.00	0.14	0.07	0.00	<0.0001
	Markazi	0.28	0.00	0.06	0.00	0.00	0.06	0.17	0.00	0.00	0.00	0.00	<0.0001
	Hormozgan	0.25	0.00	0.00	0.00	0.13	0.00	0.00	0.00	0.13	0.00	0.00	<0.0001
	Alborz	0.12	0.00	0.00	0.00	0.04	0.04	0.04	0.00	0.00	0.00	0.00	<0.0001
	Khuzestam	0.07	0.00	0.00	0.00	0.00	0.00	0.00	0.05	0.02	0.00	0.00	<0.0001
	Semnan	0.32	0.00	0.00	0.00	0.00	0.00	0.00	0.32	0.00	0.00	0.00	<0.0001
	Kohkiloye boyer ahmad	0.3	0.00	0.15	0.15	0.00	0.00	0.00	0.00	0.00	0.00	0.00	<0.0001
	Esfahan	0.02	0.00	0.00	0.00	0.00	0.00	0.00	0.00	0.02	0.00	0.00	<0.0001
	Lorestan	0.06	0.00	0.03	0.00	0.00	0.03	0.00	0.00	0.00	0.00	0.00	<0.0001
**Gender**	**Gender**	**Incidence Rate per 100,000 pop**			***p*-value**		**Occupation**		**Occupation**		**No. of Cases**	***p*-value**	
	Female	4.09			<0.0001								
	Male	9.05			<0.0001				Paddy farmers		1895	<0.0001	
									Other farmers		291	<0.0001	
**Age**	**Age Group**	**No. of Cases**		**Number of deaths**	***p*-value**				Rancher		34	<0.0001	
									Slaughterhouse workers		8	<0.0001	
	0-14	27		1	<0.0001								
	15-34	782		10	<0.0001				Students		66	<0.0001	
	35-64	2204		23	<0.0001				Military personnel		13	<0.0001	
	65<	402		12	<0.0001				Other		1126	<0.0001	

**Fig. 3:**
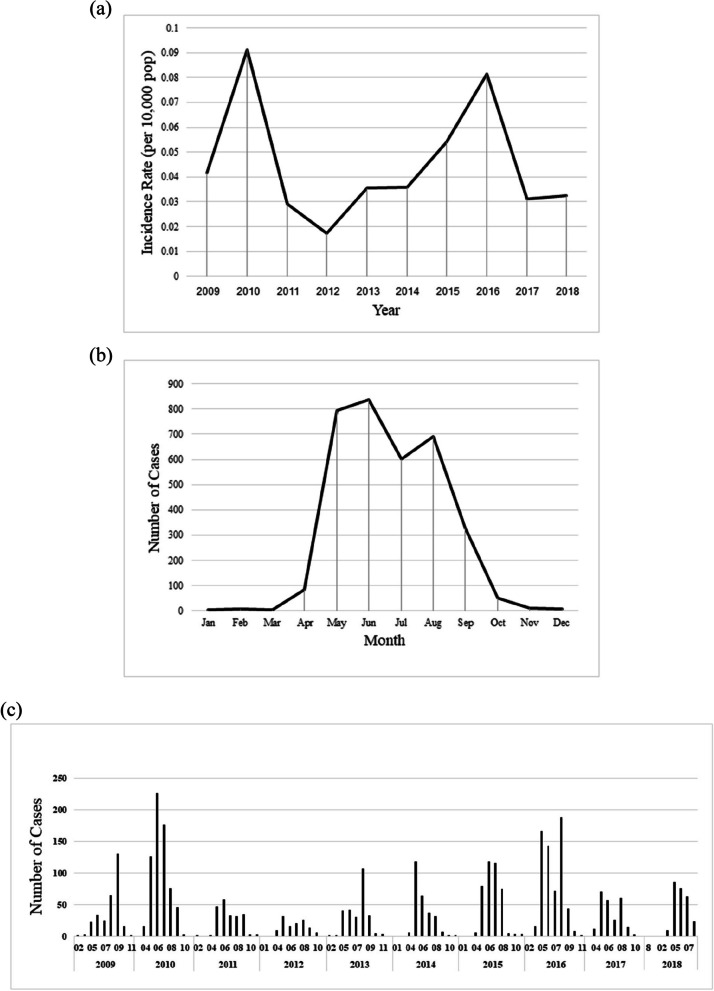
Illustrating Leptospirosis **a** yearly incidence rate, **b** total monthly number of cases and **c** time series number of cases in Iran from 2009 to 2018

 Figure 3a depicts the incidence rate for each year Fig. 3b, the total number of cases for each month during the entire study period Fig. 3c time series number of cases. Leptospirosis was prevalent in certain months of the year, from May to June, during the agricultural season. Also, it is clear from Table 1. that the number of paddy farmers is significantly higher than others during the months of May and July to August and October.

### Spatial and temporal analysis

**Fig. 4:**
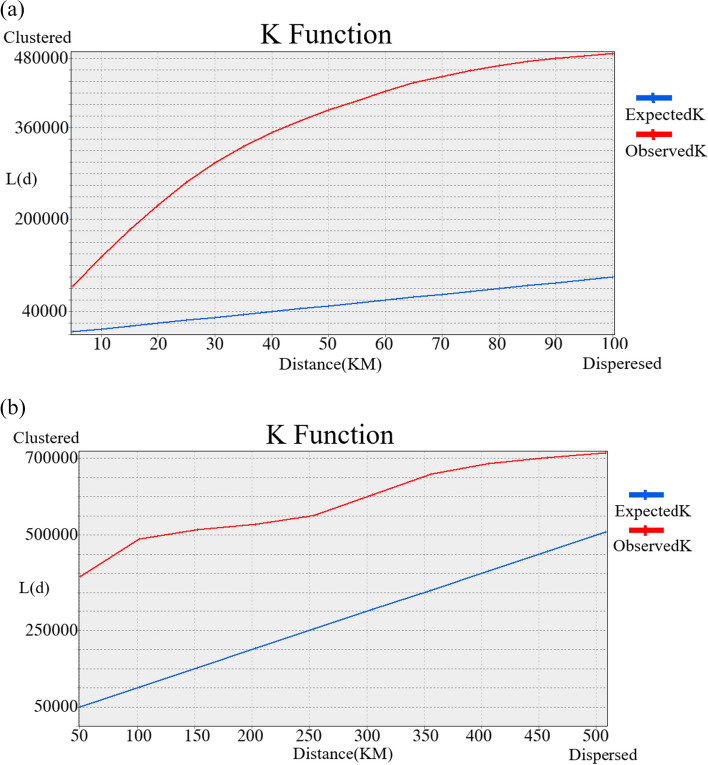
Illustrating **a** Ripley ‘K function results for distances up to 100 km and **b** all distances in Iran

 The Ripley’s K function analysis, encompassing distances up to 100 km from cases and all distances within the study area, highlights a significant finding. The observed K function (red line) surpasses the expected K function (blue line), indicating a notable statistical significance (*P*-value < 0.01) in the distribution of Leptospirosis cases in Iran Fig. 4. Evidently, the majority of occurrence points are clustered, rather than being randomly distributed.

**Fig. 5:**
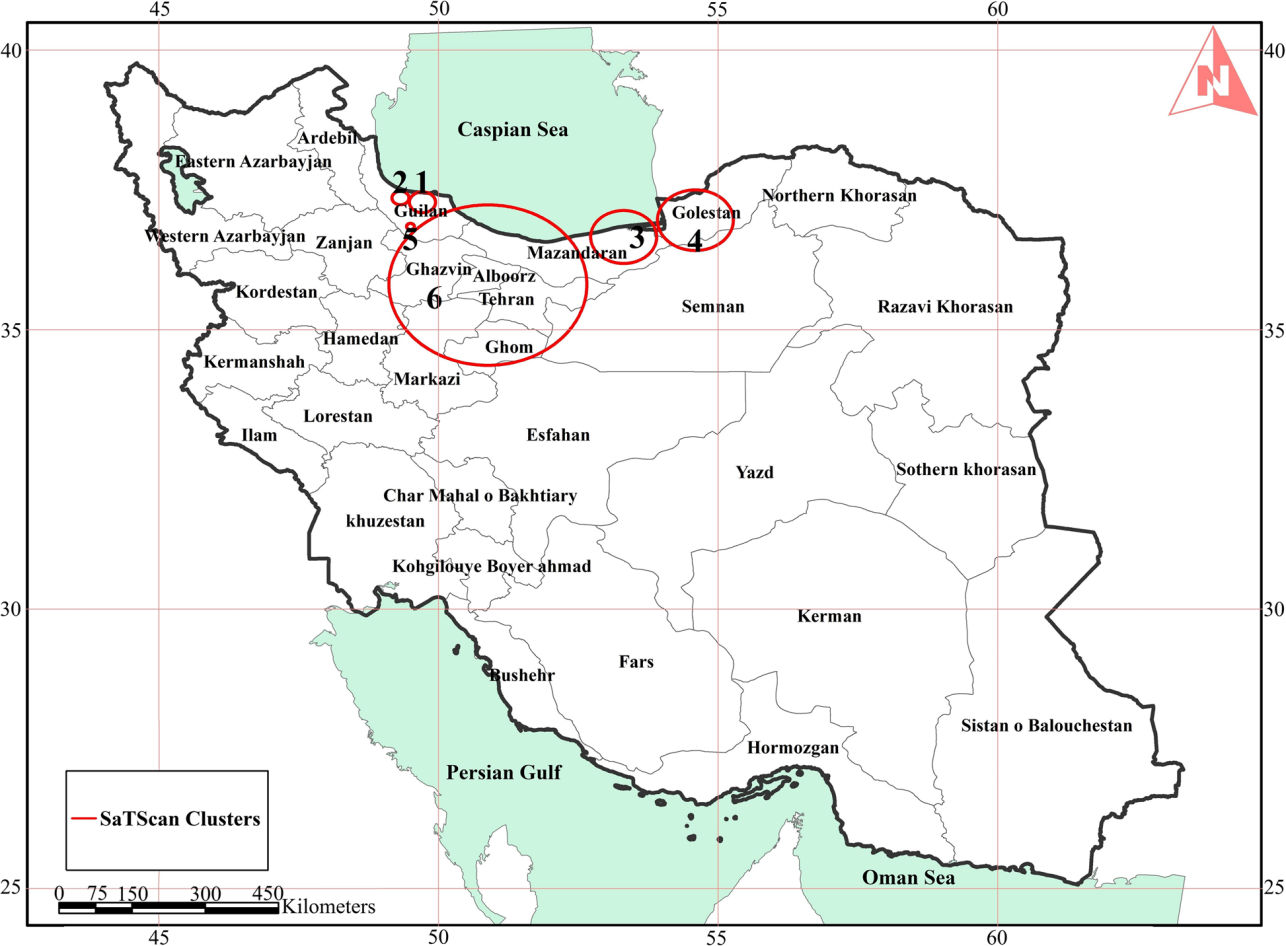
Spatial clusters of Leptospirosis detected by spatial scan statistics (SaTScan) in Iran 2009–2018

Figure 5 illustrates the identification of six distinct spatial clusters of Leptospirosis incidents spanning from 2009 to 2018. Interesting, these clusters extend beyond the previously presumed focal areas of the three northern Iranian provinces. Furthermore, a notable cluster with a radius of 159 km encompasses significant portions of provinces including Mazandaran, Gilan, Tehran, Alborz, Qazvin, and Qom. Seeking medical attention in the capital or potentially contracting the infection during trips to high-risk areas may lead to some unexpected records in certain provinces like Tehran within the clusters. Table 2 contains more details about location and time period of these six clusters. A cluster is statistically significant when its test statistic is greater than the critical value. In that way the first cluster near Rasht is the one which has the highest statistical significance with 64.34 test statistics value.

**Table 2:** Illustrating temporal and spatial clusters in Leptospirosis cases in Iran from 2009 to 2018

**Cluster ID**	**Nearby populated area to center**	**Radius (Km)**	**Start time**	**End time**	**Number of cases**	**Expected cases**	**Test statistics**	***P*-value**
**1**	Rasht	21	2013-07	2015-07	247	111.99	64.34	<0.0001
**2**	Someh Sara	13	2009-05	2011-05	190	80.01	56.93	<0.0001
**3**	Neka	53	2015-08	2018-08	273	137.61	55.67	<0.0001
**4**	Gorgan	60	2009-10	2010-04	24	2.23	35.31	<0.0001
**5**	Rudbar	6	2016-06	2016-07	10	0.67	17.67	<0.001
**6**	Karaj	159	2017-09	2018-04	11	1.44	12.9	<0.05

### Spatial disease modeling

The covariates’ maps include, mean temperature (see Additional file 1: Appendix Fig. A.1.a), maximum temperature (see Additional file 1: Appendix Fig. A.1.b), precipitation (see Additional file 1: Appendix Fig. A.1.c), NDVI (see Additional file 1: Appendix Fig. A.2), land cover (see Additional file 1: Appendix Fig. A.3), water areas (see Additional file 1: Appendix Fig. A.4.a), displacement areas (see Additional file 1: Appendix Fig. A.4.b), DEM (see Additional file 1: Appendix Fig. A.5.a), and slope (see Additional file 1: Appendix Fig. A.5.b) were carefully prepared to ensure consistent pixel dimensions and alignment across the study area. We have used a 4-fold cross-validation with RMSE as a metric of accuracy. The cross-validation results for the Inverse Distance Weighting (IDW) method applied to climate data are promising, underscoring IDW’s robust capability to generate continuous climatic data for the region using Iran’s synoptic centers. Specifically, the cross-validation values for maximum temperature, mean temperature, and precipitation are 2.94, 2.71, and 194.2 respectively.

**Fig. 6:**
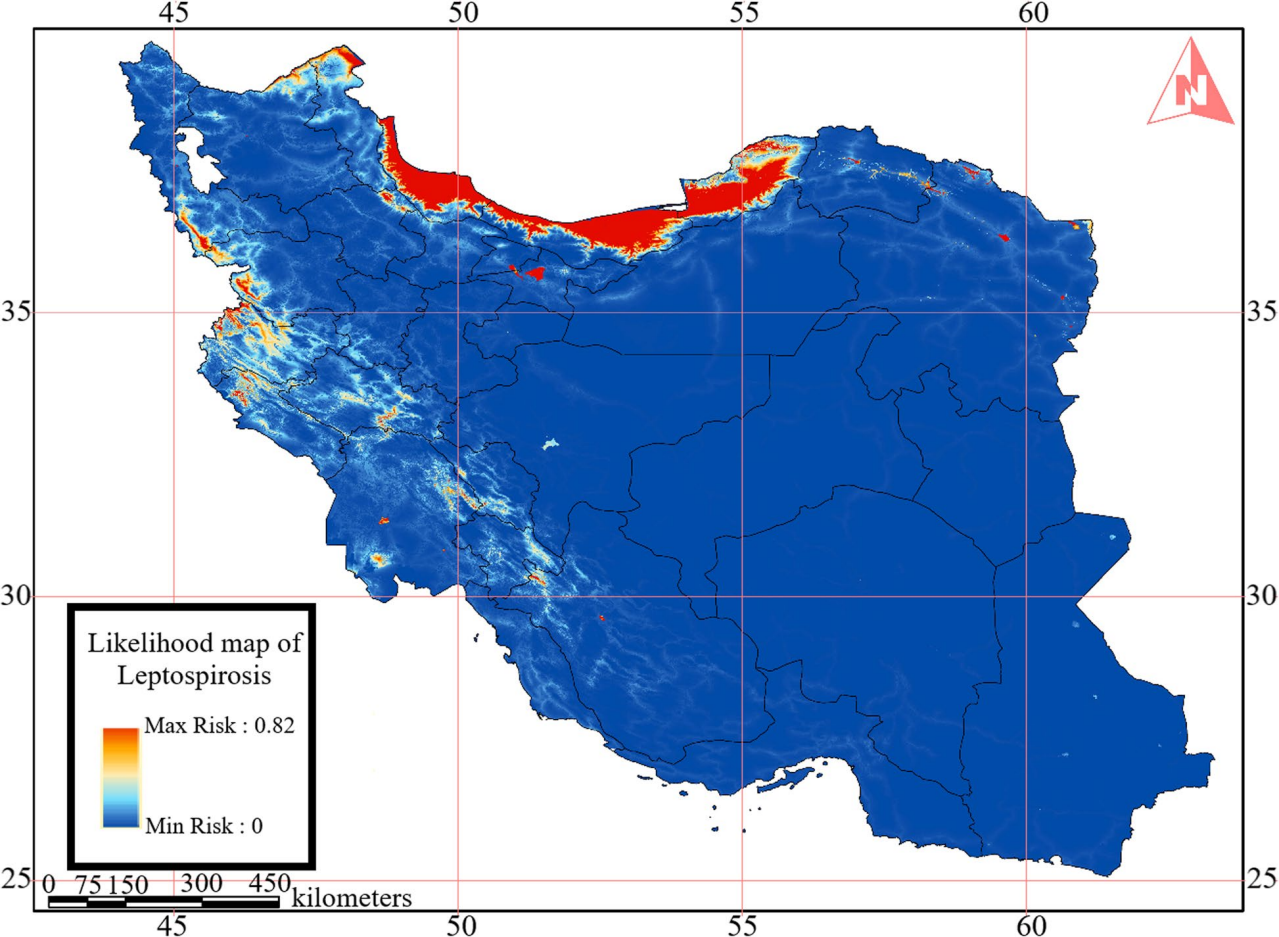
Likelihood map of Leptospirosis in Iran as a result of MaxEnt modeling

Figure 6 illustrates the MaxEnt model’s output, presenting the likelihood of Leptospirosis disease occurrence. This map integrates disease cases reported from 2009 to 2018 with environmental factors. The proximity of pixel values to 1 signifies a higher likelihood of occurrence. The risk values within this map cover a range from 0 to 0.82.

**Fig. 7:**
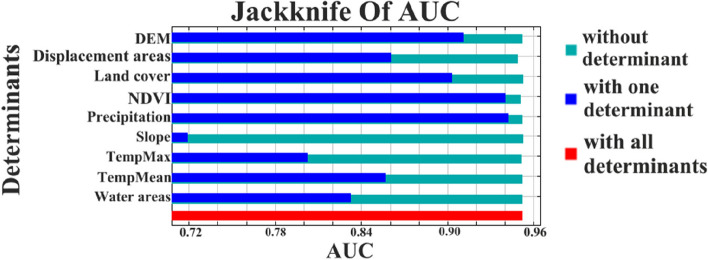
Jackknife of AUC for Leptospirosis cases and determinants

The findings highlight that in addition to the three northern provinces that are established as the epicenters of this disease in Iran, certain northern regions within Ardabil province and western provinces have also emerged as disease-prone areas. Notably, MaxEnt’s AUC metrics for training and test data yielded values of 0.956 and 0.952, respectively, underscoring the robust accuracy of the implemented model. The model’s output (see Table 3) underscores that altitude and rainfall significantly contribute to the existing distribution of Leptospirosis in Iran. Moreover, the Jackknife’s method identifies precipitation as the most pivotal determinant influencing the AUC of the outcome (see Fig. 7).

**Table 3:** Percentage of determinants’ contributions calculated by the MaxEnt model

**Determinant**	**Contributions (%)**
DEM	**41.2**
NDVI	**6.9**
Precipitation	**32.5**
Mean Temperature	**1.6**
Max Temperature	**0.3**
Slope	**0.3**
Displacement Areas	**1.2**
Water Areas	**0**
Land Cover	**16.1**

**Fig. 8:**
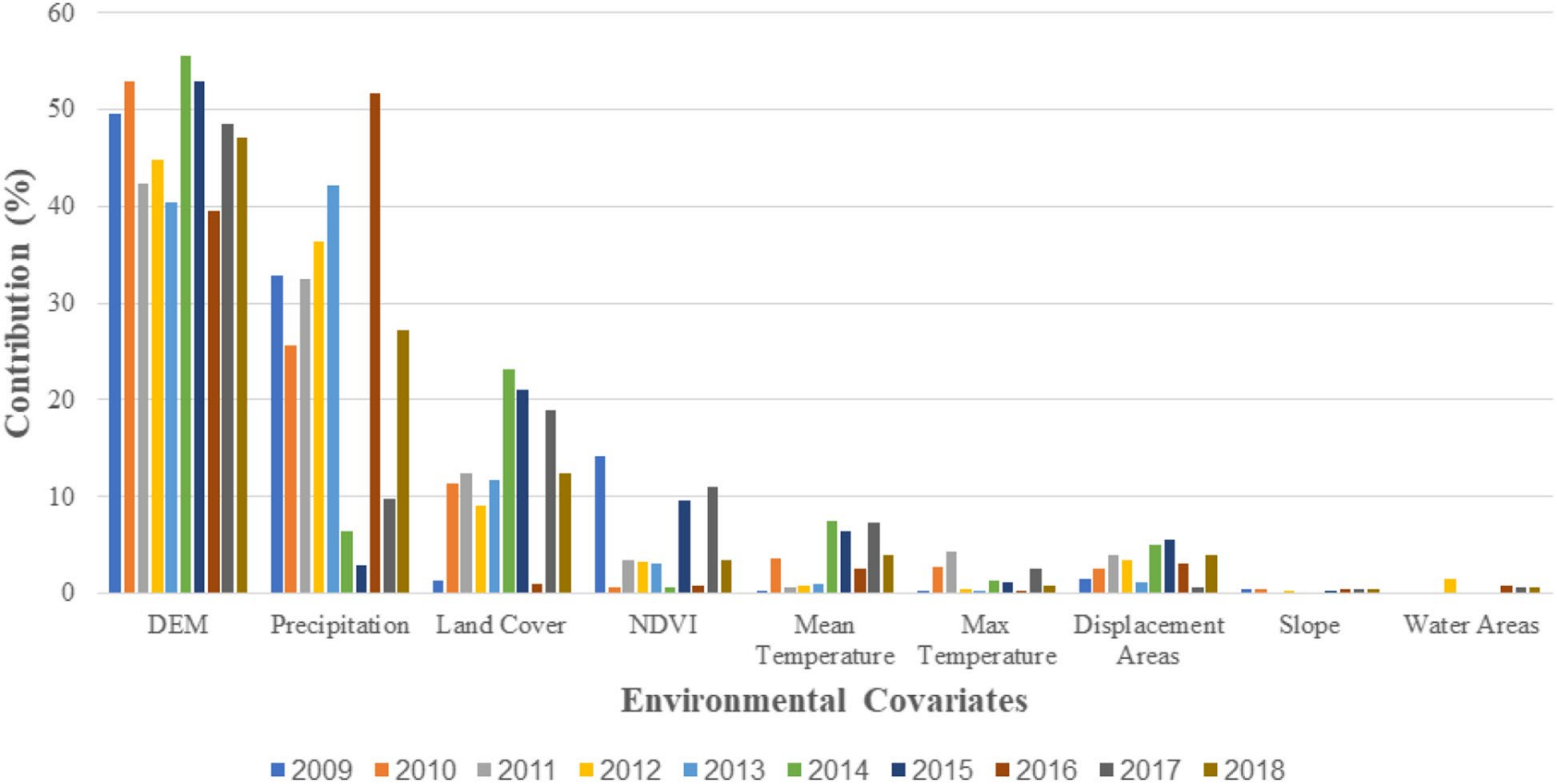
Illustrating percentage of environmental determinants’ contribution in annual MaxEnt models

Figure 8 depicts the contribution of determinants in annual models which examine the associations between environmental data in each year and disease statistics in the same year.

**Fig. 9:**
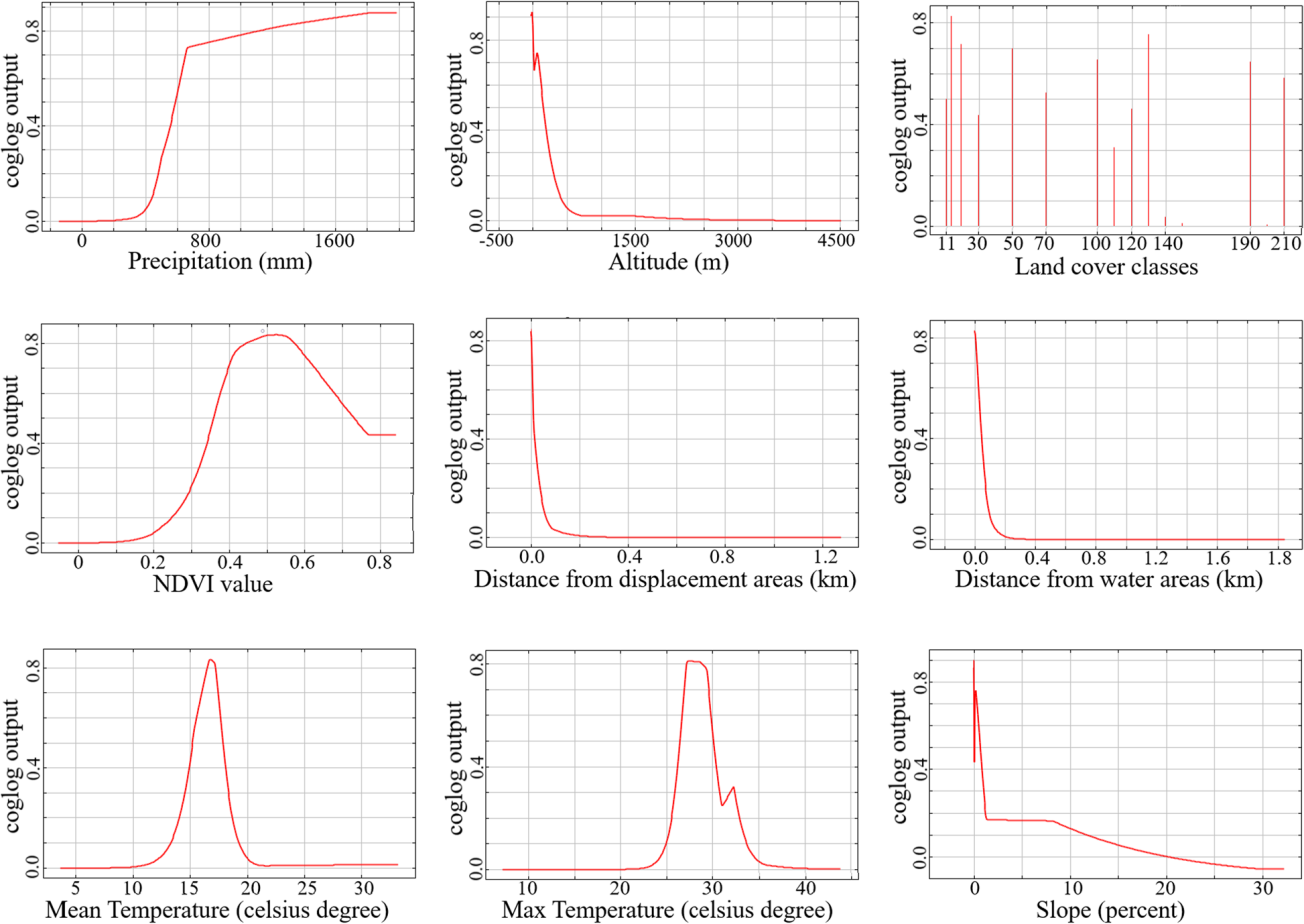
Response of the model to the nine input environmental determinants

 Figure 9 depicts how changes in the values of nine input determinants in the model can affect the risk of Leptospirosis prevalence. Precipitation positively correlates with the risk of prevalence Fig. 9a. In contrast, altitude, distance from displacement areas, distance from water areas, and slope Fig. 9b, e, f and i have a negative correlation with the likelihood of Leptospirosis prevalence. The NDVI, mean, and maximum temperature graphs in Fig. 9d, g and h exhibit a rising pattern towards a peak value, followed by a subsequent decline. The linear progression from negative values on the x-axis to the zero point in the diagrams of Fig. 9a and d results from extrapolation beyond available data. This behavior arises as MaxEnt defaults to clamping predictions within the range of values predicted for the lowest and highest sample values of the predictor.

## Discussion

Through years of investigation, we have established that three northern provinces in Iran hold the highest vulnerability to Leptospirosis risk, aligning with existing literature [17–19]. Moreover, specific cities in the west and northern regions of Ardabil province have been recognized as prone to Leptospirosis. Additionally, certain areas within Tehran province have been highlighted as experiencing a heightened risk of disease transmission.

The prevalence of Leptospirosis is shaped by intricate interplays among environmental factors, climate, altitude, water bodies, residents’ socioeconomic statuses such as occupation, and the presence of domestic, wild, and rodent animals in the area [7]. The results obtained from MaxEnt modeling (Fig. 9) offer insights into alterations in environmental determinants that could impact the likelihood of the disease’s prevalence.

Our findings indicate that an annual precipitation exceeding 600 mm escalates the risk of prevalence. Other research corroborates this, demonstrating that heightened rainfall leads to an elevated Leptospirosis prevalence due to increased water volumes, raising the chances of contact between animal hosts and humans with contaminated water [3, 8, 13, 15, 37–39].

Additionally, the findings demonstrate that the “altitude” factor significantly influences disease prevalence when it hovers around zero and approximately 500 m. For instance, regions situated under 200 m above sea level exhibit a 50% likelihood of disease prevalence. These findings harmonize with existing studies that emphasize the substantial role of altitude in Leptospirosis prevalence [17, 18, 40], with a higher frequency of occurrence noted at lower altitudes [17, 40, 41]. Furthermore, “altitude” contributes around 40% to all annual models, signifying a pronounced reliance of the disease on this factor. The outcomes also indicate that flat terrains are conducive to disease prevalence, a correlation supported by a study conducted in northern Iran [18].

The third noteworthy determinant in our model is “land cover,“ contributing 16.1% to our model and acknowledged as a significant factor in Leptospirosis prevalence [3, 15, 37, 39]. More than 70% of the disease prevalence affecting paddy farmers can be attributed to rainfed croplands, mosaic cropland, and vegetation classes. Furthermore, evergreen or deciduous forests are two other impactful land covers influencing Leptospirosis prevalence, consistent with prior research findings [15]. These areas conducive to animals can expose hosts to infected carrier animals and their habitats [13, 15]. While a study in China [3] indicated that “mean temperature” holds more influence than “land cover,“ our study demonstrates that “land cover” holds greater influence than “mean temperature” in modeling Leptospirosis in Iran.

The model’s response to the “NDVI” factor indicates that, for the occurrence of Leptospirosis, the area should not be devoid of vegetation, even if it is sparse, and it should also not be dense. The “NDVI” contribution percentage varies across yearly models. A study conducted in the United States found that the relationship between vegetation and animal Leptospirosis is uncertain, possibly due to the effects of different vegetation types on disease prevalence [15]. As a result, areas with moderate vegetation levels provide the best conditions for the spread of Leptospirosis.

The variable “displacement areas” was incorporated to explore the impact of proximity to roads and entry border points on the incidence of Leptospirosis. This variable introduces a novel perspective, as it is the first time it’s been used in a Leptospirosis model to investigate the role of human and animal movement in bacteria spread and transmission. For instance, the movement of humans and the export of items like flowers and plants, notably bamboo, play crucial roles in the expansion of vectors for diseases like Chikungunya to new regions [42]. Our study findings unveil that while the displacement area variable contributes only around 1% to Leptospirosis prevalence, it holds greater significance than determinants like maximum temperature, slope, and surface water. This determinant has been recognized as an influential factor in disease vector transmission [42, 43].

Furthermore, the Jackknife analysis underscores the significance of the “displacement areas” factor, given that its removal results in the most pronounced decrease in model accuracy. The findings from “distance from displacement areas” and “distance from water” variables suggest that the disease exhibits higher prevalence in areas below 100 m. Given that contact with contaminated water is a primary route of Leptospirosis transmission [3, 13, 37], the impact of “water areas” on disease prevalence seems to be restricted. However, considering that Leptospirosis is more prevalent in areas with inadequate urban sanitation and rural environments [13], it is indicated that disease prevalence is not closely tied to high-volume water bodies like rivers and lakes [3]. Instead, the source of Leptospirosis transmission is likely polluted water in cavities or fields [3].

The findings reveal that an average temperature ranging from approximately 15 to 20 degrees Celsius, along with a monthly maximum temperature of about 15 to 25 degrees Celsius per year, creates the most favorable conditions for prevalence. While certain studies have cited temperature as a pivotal factor in Leptospirosis prevalence [3, 41], our analysis indicates that both mean and maximum temperatures do not significantly influence the modeling of Leptospirosis in Iran.

Additionally, the prevalence of leptospirosis in middle-aged adults in Iran is more than twice that of all other age groups, which is consistent with reported cases in Mexico [14]. Furthermore, among males, the epidemy was twice as associated with occupation, particularly in the rice industry. Farmers in Iran come into direct contact with surface water while barefoot and barehanded, increasing the likelihood of direct contact with the disease pathogen and, as a result, the disease’s prevalence [19, 44]. Furthermore, more than half of people in the occupational category’s “other” subcategory (Table 1) report having had contact with contaminated water, swimming in a river, or drinking non-tap water. As a result, water recreation in hot weather could create the conditions for this bacterium to be transmitted to the host.

## Strengths and limitations

There are a few limitations to this study. Socioeconomic and demographic data (e.g., poverty rate, grided population maps, educational level) may contribute to produce more accurate results [3, 13, 40]. However, due to data unavailability and accessibility constraints, we were unable to incorporate them. Additionally, the absence of systematic recording for Leptospirosis absence data in Iran posed a challenge. Consequently, we utilized randomly generated pseudo-absence data. Employing absence locations serves to mitigate the impact of random pseudo-absences, enhancing result accuracy and preventing model overfitting. Furthermore, Iran’s surveillance system operates as a passive entity, potentially leading to an underestimation of recorded cases. Consequently, there exists a propensity for official disease statistics to be underestimated. It is worth noting that discrepancies could exist between recorded residential addresses, used as disease case locations, and the actual infection sites.

## Conclusion

The spatio-temporal modeling approach serves as a valuable tool for comprehending the distribution of Leptospirosis and its influential determinants. Through this method, significant clusters of Leptospirosis have been identified in Iran over the years, particularly in areas such as Gilan province and the eastern Caspian Sea region, as well as Tehran and Alborz provinces. Furthermore, regions with a susceptibility to Leptospirosis prevalence are notable in the northwest and west of the country. Among the determinants considered, precipitation and altitude have emerged as two critical factors with substantial impact in delineating the disease’s risk areas across Iran. Given the escalating global burden of Leptospirosis, future research endeavors should encompass a broader spectrum of determinants, including demographic and socioeconomic variables, to better identify vulnerable populations. Understanding the spatial and temporal dynamics of emerging infectious and zoonotic diseases holds immense significance for predicting outbreaks and devising effective interventions. Hence, the exploration of space-time interactions is highly recommended for advancing our knowledge in this field.

### Supplementary Information

**Additional file 1**: **Appendix Fig.A.1.** (a) Average temperature, (b) average maximum monthly temperature and (c) average yearly precipitation maps in Iran from 2009-2018. **Appendix Fig.A.2.** Average Normaized Differenece Vegetation Index (NDVI) map in Iran from 2009-2018. **Appendix Fig.A.3.** Map of Landcover in Iran based on GlobeCover map. **Appendix Table A.1.** Description of different classes of Landcover map. **Appendix Fig.A.4. **(a) Maps of distance from water areas and (b) displacement areas. **Appendix Fig.A.5. **(a) DEM and (b) Slope maps of the study area.

## Data Availability

The datasets used and/or analysed during the current study are available from the corresponding author on reasonable request.
