# Supplementary material for: Spatio-temporal modeling of human leptospirosis prevalence using the maximum entropy model
Source: BMC Public Health. 2023 Dec 16;23:2521. doi: 10.1186/s12889-023-17391-z (PMC10724969; doi:10.1186/s12889-023-17391-z)
Supplement: Supplementary file 1 — Additional file 1: Appendix Fig.A.1. (a) Average temperature, (b) average maximum monthly temperature and (c) average yearly precipitation maps in Iran from 2009-2018. Appendix Fig.A.2. Average Normaized Differenece Vegetation Index (NDVI) map in Iran from 2009-2018. Appendix Fig.A.3. Map of Landcover in Iran based on GlobeCover map. Appendix Table A.1. Description of different classes of Landcover map. Appendix Fig.A.4. (a) Maps of distance from water areas and (b) displacement areas. Appendix Fig.A.5. (a) DEM and (b) Slope maps of the study area. [file 12889_2023_17391_MOESM1_ESM.docx]

**Appendix A**

| (a)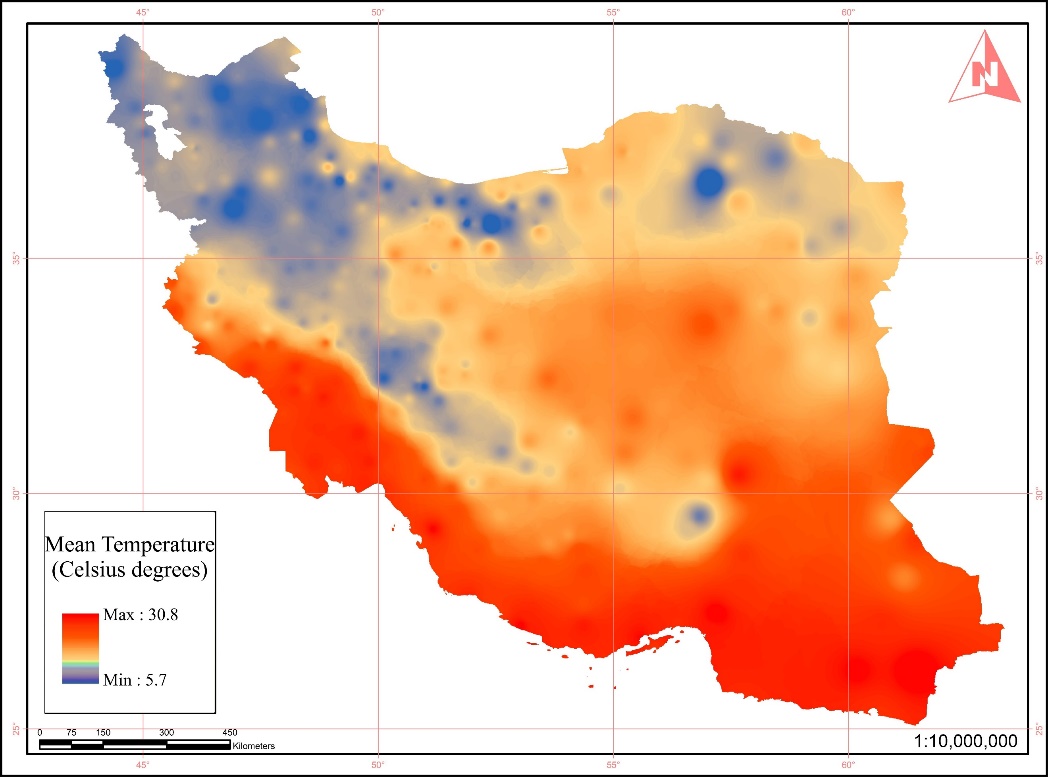 | (b)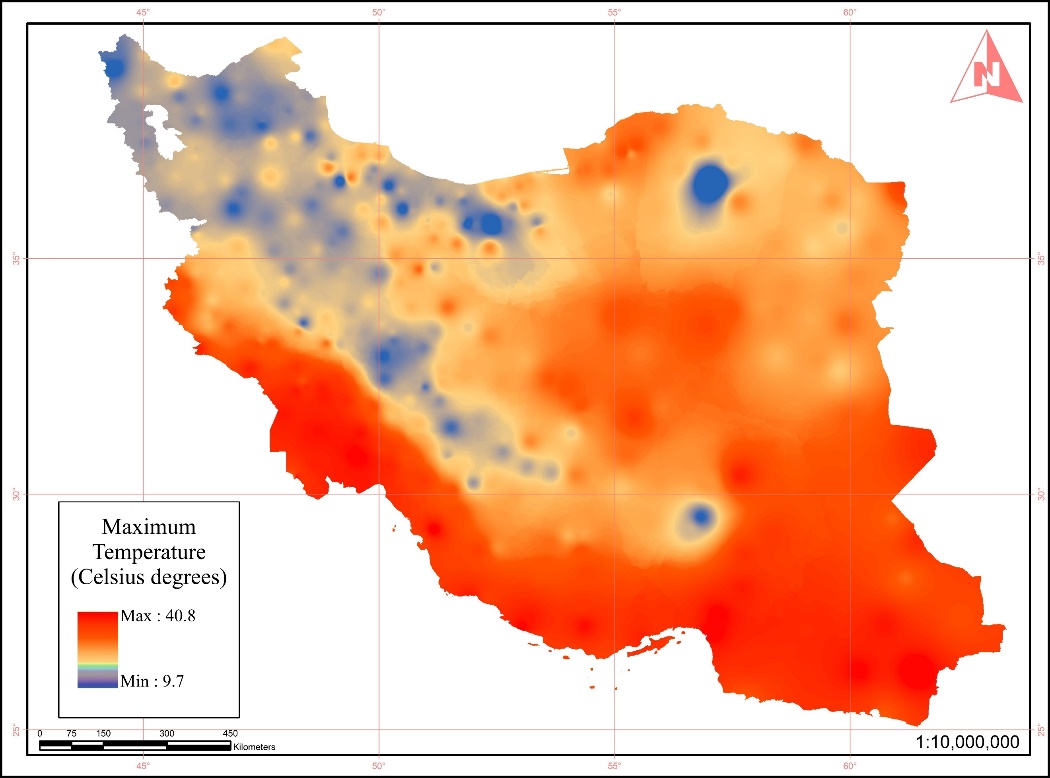 |
| --- | --- |
| (c) |  |
| 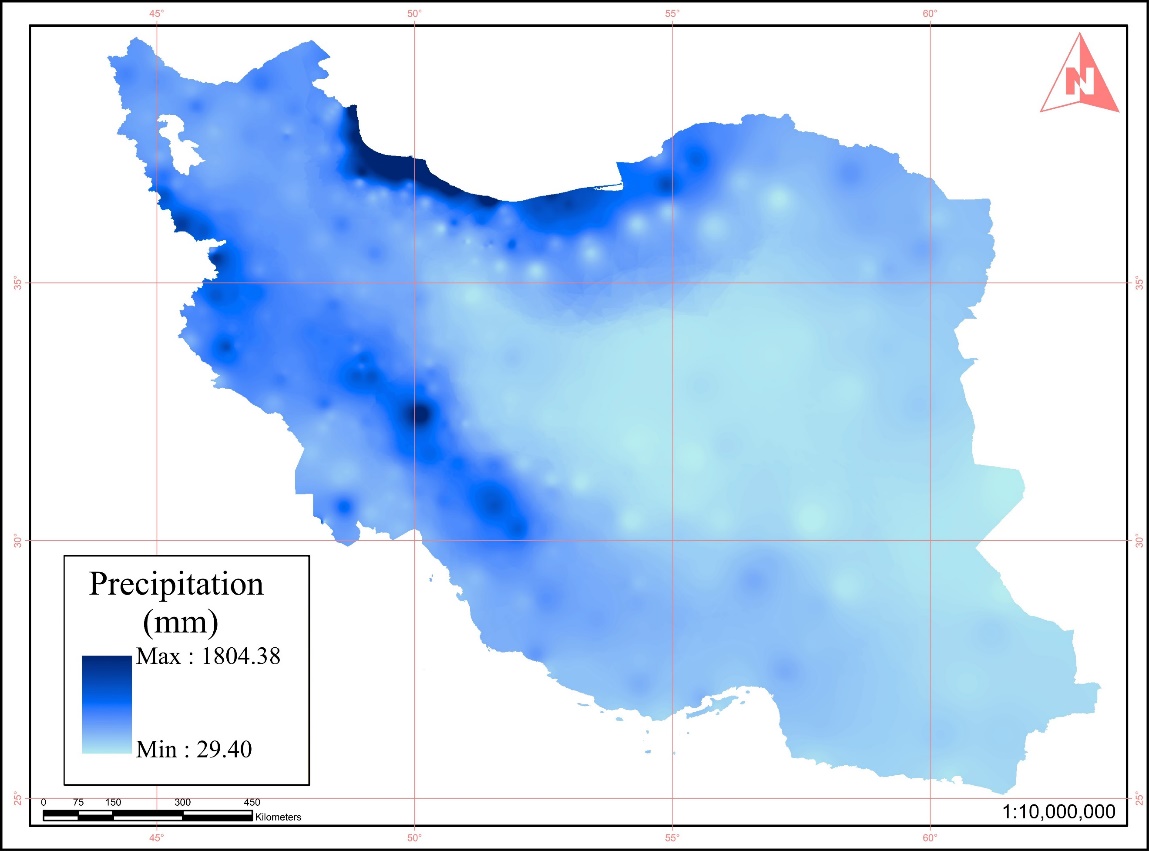 | |
| **Appendix Fig.A.1.** (a) Average temperature, (b) average maximum monthly temperature and (c) average yearly precipitation maps in Iran from 2009-2018. | |


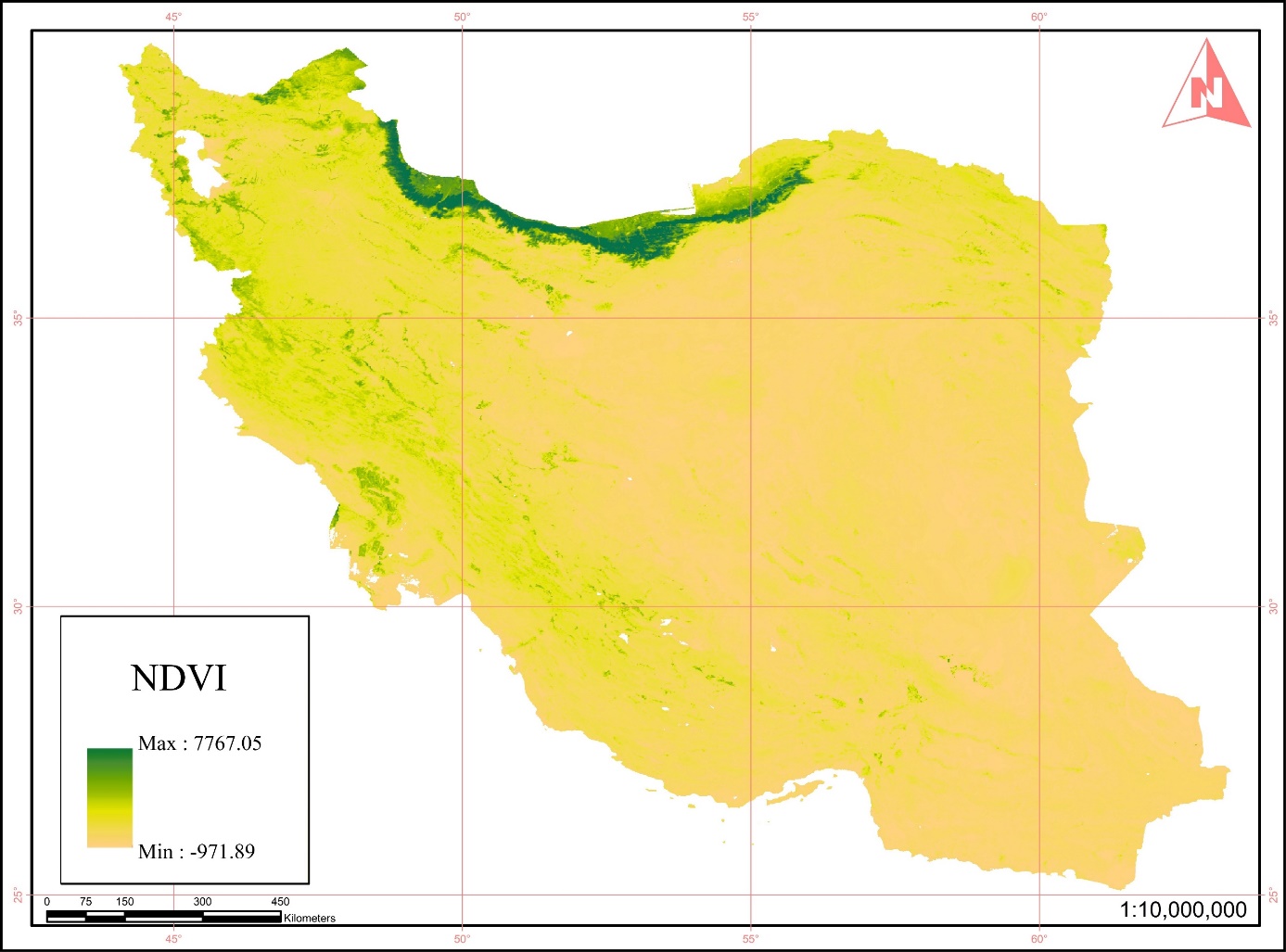


**Appendix Fig.A.2.** Average Normaized Differenece Vegetation Index (NDVI) map in Iran from 2009-2018

***
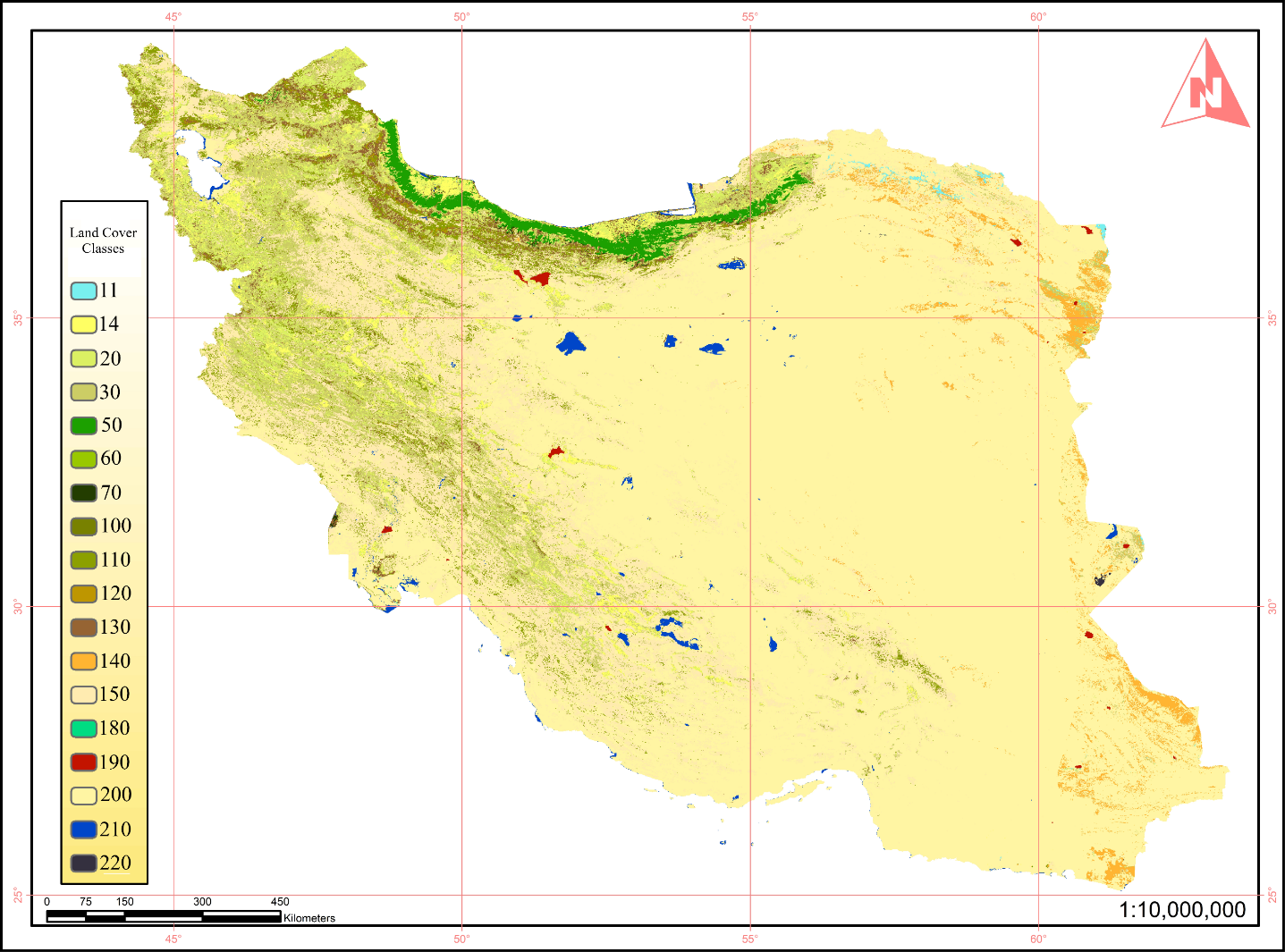
***

**Appendix Fig.A.3.** Map of Landcover in Iran based on GlobeCover map.

**Appendix Table A.1.** Description of different classes of Landcover map

| Class | Description |
| --- | --- |
| 11 | Post-flooding or irrigated croplands (or aquatic) |
| 14 | Rainfed croplands |
| 20 | Mosaic cropland (50-70%) / vegetation (grassland/shrubland/forest) (20-50%) |
| 30 | Mosaic vegetation (grassland/shrubland/forest) (50-70%) / cropland (20-50%) |
| 40 | Closed to open (>15%) broadleaved evergreen or semi-deciduous forest (>5m) |
| 50 | Closed (>40%) broadleaved deciduous forest (>5m) |
| 60 | Open (15-40%) broadleaved deciduous forest/woodland (>5m) |
| 70 | Closed (>40%) needleleaved evergreen forest (>5m) |
| 90 | Open (15-40%) needleleaved deciduous or evergreen forest (>5m) |
| 100 | Closed to open (>15%) mixed broadleaved and needleleaved forest (>5m) |
| 110 | Mosaic forest or shrubland (50-70%) / grassland (20-50%) |
| 120 | Mosaic grassland (50-70%) / forest or shrubland (20-50%) |
| 130 | Closed to open (>15%) (broadleaved or needleleaved, evergreen or deciduous) shrubland (<5m) |
| 140 | Closed to open (>15%) herbaceous vegetation (grassland, savannas or lichens/mosses) |
| 150 | Sparse (<15%) vegetation |
| 160 | Closed to open (>15%) broadleaved forest regularly flooded (semi-permanently or temporarily) – Fresh or brackish water |
| 170 | Closed (>40%) broadleaved forest or shrubland permanently flooded - Saline or brackish water |
| 180 | Closed to open (>15%) grassland or woody vegetation on regularly flooded or waterlogged soil – Fresh, brackish or saline water |
| 190 | Artificial surfaces and associated areas (Urban areas >50%) |
| 200 | Bare areas |
| 210 | Water bodies |
| 220 | Permanent snow and ice |
| 230 | No data (burnt areas, clouds, …) |

| (a) | (b) |
| --- | --- |
| 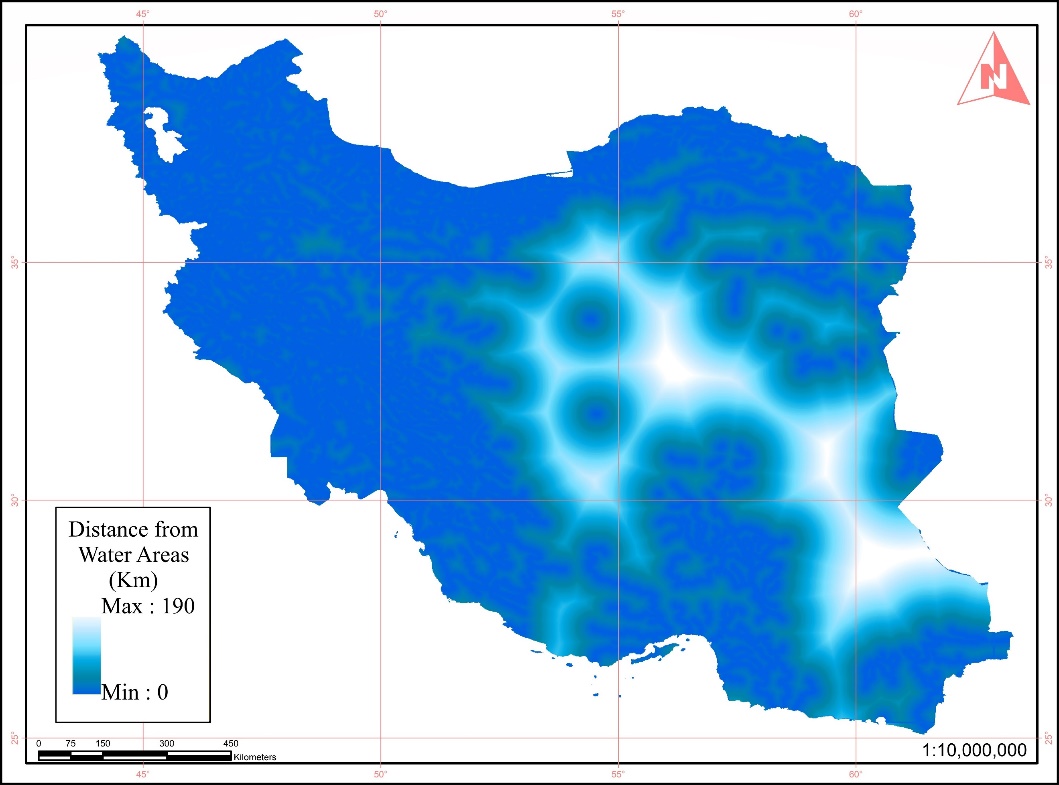 | 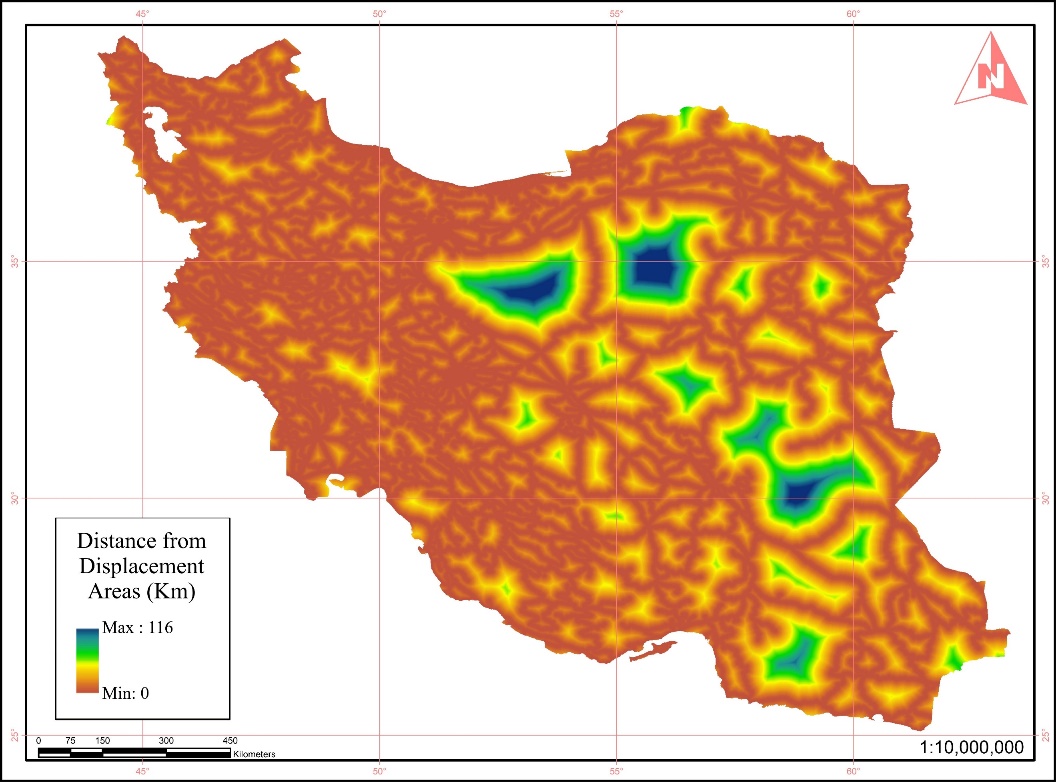 |

**Appendix Fig.A.4.** (a) Maps of distance from water areas and (b) displacement areas.

| (a) | (b) |
| --- | --- |
| 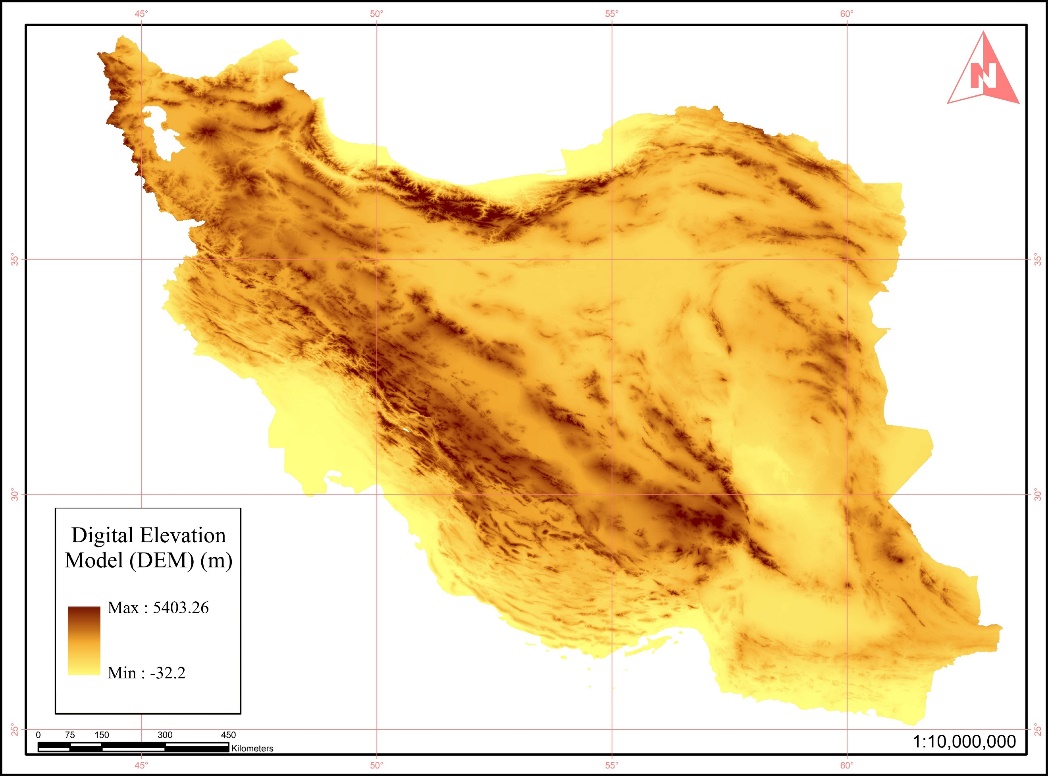 | 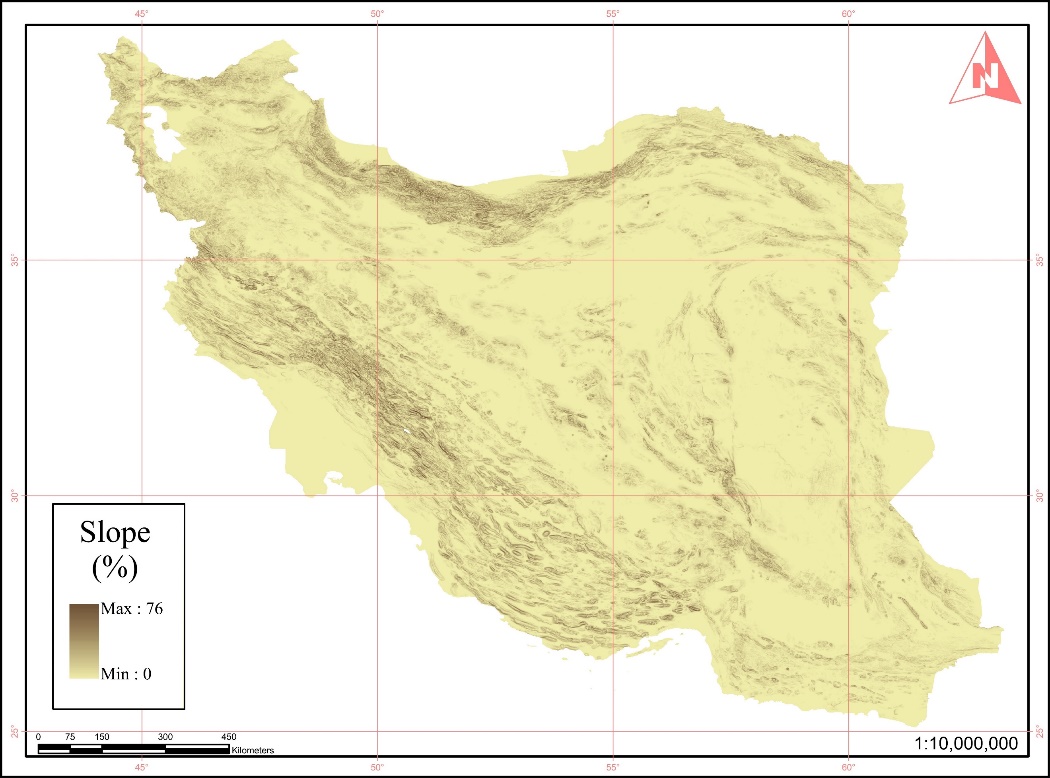 |

**Appendix Fig.A.5.** (a) DEM and (b) Slope maps of the study area.
